# Newspaper Signs

**Published:** 1858-03

**Authors:** 


### NEWSPAPER SIGNS.

THE march of improvement has of late years manifested itself in almost every department of human life, but none has been made to the old-fashioned way of travel on horseback; for it is the most instructive, and the most healthful, as well as the most delightful of all. In the far-back years of the past, we had large experience of that kind, and early learned to select a "stopping-place" from external appearances; the most reliable "sign" being flowers and flower-pots about the doors and windows, for there is sure to be within, a woman of taste, tidiness, and character.

In passing through the city, or entering houses for the first time, we find ourselves deciding upon the character of the inmates from the newspapers we see at the door, and the periodicals lying about; and we feel a guarantee that there is refinement and elevation within, when we see *The Home Journal*, *The Scientific American*, *The Musical World*, *Littell's Living Age*, and publications of that stamp. These four might be profitably taken by every family in New York, and ought to be taken in thousands where they are not; for they are always chaste, always instructive, nothing in them to blunt the moral sense, or offend our religious sentiment: in these, and some *one* good religious newspaper, there is as much reading of this sort as the generality of our households can profitably indulge in. And far more creditable is it, as a sign of good, to see in a young man's hand or room such a sterling periodical as *The Young Men's Magdzine*, of New York, than one of the flashy monthlies, or the inane weekly, with its trifling love-story, its criminal calendar, its levelling, and its insidiously infidel editorials.

And if, upon entering the dwelling of one of our Baptist subscribers, we should see a copy of that able and just quarterly, *The Christian Review*, of Baltimore, we should take it for granted that we were in a family of intelligent Christians, well indoctrinated, and not only so, but well trained, as to two very cardinal points in practical religion; to wit, a liberal benevolence and the sanctity of the Sabbath.

